# Complete Genome Sequence of Rhodococcus qingshengii Strain CL-05, Isolated from Concrete

**DOI:** 10.1128/MRA.00376-21

**Published:** 2021-05-13

**Authors:** E. Anders Kiledal, Shannon G. McDermott, Olga Shevchenko, Jean Ross, Stacy Bediako, Julia A. Maresca

**Affiliations:** a Department of Biological Sciences, University of Delaware, Newark, Delaware, USA; b Department of Civil and Environmental Engineering, University of Delaware, Newark, Delaware, USA; c Sequencing and Genotyping Center, University of Delaware, Newark, Delaware, USA; d BioImaging Center, University of Delaware, Newark, Delaware, USA; University of Arizona

## Abstract

Here, we report the complete genome sequence of Rhodococcus qingshengii strain CL-05, which was isolated from pavement concrete in Newark, Delaware. The genome consists of a 6.29-Mbp chromosome and one plasmid (123,183 bp), encodes a total of 5,859 predicted proteins, and has a GC content of 62.5%.

## ANNOUNCEMENT

We previously isolated several bacterial strains from concrete, an alkaline, high-salt environment (1), and then sequenced the genome of *Rhodococcus* sp. strain CL-05 to compare it with those of alkaliphilic and halophilic *Rhodococcus* species (2–4).

**FIG 1:**
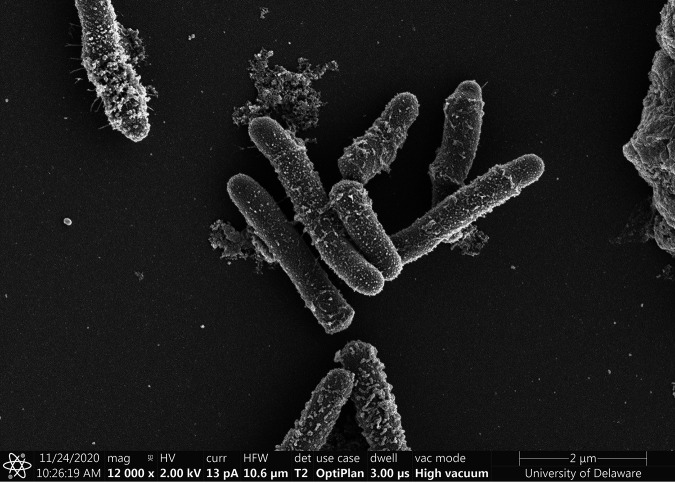
Scanning electron microscopy (SEM) of CL-05 cells. Cells were grown overnight in nutrient broth for SEM. After centrifugation, cells were resuspended in fresh 4% paraformaldehyde in phosphate-buffered saline. Suspended cells in fixative were placed onto poly-l-lysine-coated silicon wafers. After cells were allowed to attach, the sample wafers were washed with 0.1 M sodium cacodylate buffer and then incubated with 1% osmium tetroxide in 0.1 M sodium cacodylate buffer. The samples were dehydrated in a graded series of ethanol concentrations from 50% to 100%, transferred to hexamethyldisilazane, and air dried. Wafers were mounted onto SEM stubs, sputter coated with 3-nm platinum, and imaged with a Thermo Fisher Scientific Apreo SEM.

Pieces of concrete (∼1 g) were vortexed in TE buffer (10 mM Tris, 1 mM EDTA), and then 25 μl of solution was spread onto concrete medium solidified with agar (CM-A) and incubated at room temperature for ∼2 weeks (1). Individual colonies were restreaked onto CM-A until axenic, as determined by microscopy. CL-05 cells are ∼2.5- to 4-μm rods (Fig. 1), and this isolate was initially identified as Rhodococcus erythropolis by Sanger sequencing of its 16S gene (primers 8F and 1492R [1, 5]).

Strain CL-05 was revived on nutrient agar (NA) (catalog number OXCM0003; Fisher) from a −80°C stock (in 7% dimethyl sulfoxide). One colony was restreaked onto NA and grown overnight at 28°C, and then one colony was transferred to 50 ml nutrient broth and grown overnight. DNA was extracted using a phenol-chloroform extraction protocol optimized for Gram-positive bacteria (6). A single-molecule real-time (SMRT) library was barcoded and prepared using the PacBio SMRTbell Express template preparation kit version 2.0 (7). DNA fragments larger than 6 kb were size selected using BluePippin (Sage Science). The average library fragment size was 15 kb, as measured by a fragment analyzer (Advanced Analytical Technologies, Inc.). Sequencing was completed on a PacBio Sequel single-molecule sequencer in one 1M version 3 LR SMRT Cell with a 20-h movie. Samples were demultiplexed using PacBio SMRT Link version 9.0.0.92188.

We obtained 67,539 barcoded reads (size range, 51 to 91,079 nucleotides [nt]; *N*_50_, 12,320 nt). Demultiplexed raw subreads were downloaded from PacBio SMRT Link, converted to .fastq files with bam2fastx version 1.3.1, and then chimera checked, quality controlled, assembled, and circularized using Flye version 2.8.2 (8). The genome was rotated to start at *dnaA* using Circlator version 1.5.5 (fixstart method) (9). The assembly was polished using raw reads aligned with the PacBio minimap2 version 1.3.0 wrapper (10) and the Arrow polishing algorithm in pbgcpp version 1.0.0 (11). The assembled genome consists of a 6,290,587-bp circular chromosome (GC content of 62.5%, with 90× coverage) and one circular plasmid (123,183 bp; GC content of 62.4%, with 119× coverage). Default parameters were used for all software unless otherwise noted; the pipeline is available at github.com/MarescaLab/genome_pipeline.

FastANI in GTDB-Tk version 1.4.0 (12, 13) identified the closest relative of CL-05 as Rhodococcus qingshengii strain JCM 15477 (GenBank accession number GCF_001646745.1), with an average nucleotide identity (ANI) of 98.78% over 93% of the input sequence. The plasmid is >99% identical to a plasmid from R. qingshengii strain IGTS8 (GenBank accession number GCA_006384225.1).

PGAP build 5132 (14) was used to predict open reading frames and to annotate genes, using default parameters. The chromosome contains 5 rRNA operons, 59 tRNAs, 1 transfer-messenger RNA, and 5,726 predicted protein-coding genes; the plasmid contains 133 protein-coding genes.

Using BLASTp, we identified two putative Na^+^/H^+^ antiporters (*nhaA* and *mrpABCDEFG* [15, 16]) that are useful in halotolerance and alkalitolerance (17) and the putative betaine transporter *betP*, which is involved in osmoprotection (18), in the CL-05 genome.

### Data availability.

The raw reads have been submitted to the SRA and have the accession number SRR13722032. The assembled, annotated genome is available in the NCBI database under BioProject number PRJNA702129 (chromosome, accession number CP072108; plasmid, accession number CP072109).

## References

[1] Maresca JA, Moser P, Schumacher T. 2017. Analysis of bacterial communities in and on concrete. Mater Struct 50:25. doi:10.1617/s11527-016-0929-y.

[2] Mayilraj S, Krishnamurthi S, Saha P, Saini HS. 2006. *Rhodococcus kroppenstedtii* sp. nov., a novel actinobacterium isolated from a cold desert of the Himalayas, India. Int J Syst Evol Microbiol 56:979–982. doi:10.1099/ijs.0.63831-0. 16627641

[3] Goordial J, Raymond-Bouchard I, Ronholm J, Shapiro N, Woyke T, Whyte L, Bakermans C. 2015. Improved-high-quality draft genome sequence of *Rhodococcus* sp. JG-3, a eurypsychrophilic actinobacteria from Antarctic Dry Valley permafrost. Stand Genomic Sci 10:61. doi:10.1186/s40793-015-0043-8. 26380646 PMC4572675

[4] Hjerde E, Pierechod MM, Williamson AK, Bjerga GEK, Willassen NP, Smalås AO, Altermark B. 2013. Draft genome sequence of the actinomycete *Rhodococcus* sp. strain AW25M09, isolated from the Hadsel Fjord. Genome Announc 1:e0005513. doi:10.1128/genomeA.00055-13. 23516194 PMC3593323

[5] Turner S, Pryer KM, Miao VP, Palmer JD. 1999. Investigating deep phylogenetic relationships among cyanobacteria and plastids by small subunit rRNA sequence analysis. J Eukaryot Microbiol 46:327–338. doi:10.1111/j.1550-7408.1999.tb04612.x. 10461381

[6] Kiledal EA, Maresca JA. 2021. Chromosomal DNA extraction from Gram-positive bacteria. protocolsio doi:10.17504/protocols.io.bs7knhkw.

[7] Pacific Biosciences of California, Inc. 2020. Preparing multiplexed microbial libraries using SMRTbell express template prep kit 2.0. Pacific Biosciences of California, Inc., Menlo Park, CA.

[8] Kolmogorov M, Yuan J, Lin Y, Pevzner PA. 2019. Assembly of long, error-prone reads using repeat graphs. Nat Biotechnol 37:540–546. doi:10.1038/s41587-019-0072-8. 30936562

[9] Hunt M, Silva ND, Otto TD, Parkhill J, Keane JA, Harris SR. 2015. Circlator: automated circularization of genome assemblies using long sequencing reads. Genome Biol 16:294. doi:10.1186/s13059-015-0849-0. 26714481 PMC4699355

[10] Li H. 2018. minimap2: pairwise alignment for nucleotide sequences. Bioinformatics 34:3094–3100. doi:10.1093/bioinformatics/bty191. 29750242 PMC6137996

[11] Pacific Biosciences. GCpp: generate highly accurate reference contigs. github.com/PacificBiosciences/gcpp.

[12] Jain C, Rodriguez-R LM, Phillippy AM, Konstantinidis KT, Aluru S. 2018. High throughput ANI analysis of 90K prokaryotic genomes reveals clear species boundaries. Nat Commun 9:5114. doi:10.1038/s41467-018-07641-9. 30504855 PMC6269478

[13] Chaumeil P-A, Mussig AJ, Hugenholtz P, Parks DH. 2019. GTDB-Tk: a toolkit to classify genomes with the Genome Taxonomy Database. Bioinformatics 36:1925–1927. doi:10.1093/bioinformatics/btz848. PMC7703759 31730192

[14] Tatusova T, DiCuccio M, Badretdin A, Chetvernin V, Nawrocki EP, Zaslavsky L, Lomsadze A, Pruitt KD, Borodovsky M, Ostell J. 2016. NCBI Prokaryotic Genome Annotation Pipeline. Nucleic Acids Res 44:6614–6624. doi:10.1093/nar/gkw569. 27342282 PMC5001611

[15] Huang Y, Chen W, Dotson DL, Beckstein O, Shen J. 2016. Mechanism of pH-dependent activation of the sodium-proton antiporter NhaA. Nat Commun 7:12940. doi:10.1038/ncomms12940. 27708266 PMC5059715

[16] Fang H, Qin X-Y, Zhang K-D, Nie Y, Wu X-L. 2018. Role of the group 2 Mrp sodium/proton antiporter in rapid response to high alkaline shock in the alkaline- and salt-tolerant *Dietzia* sp. DQ12-45-1b. Appl Microbiol Biotechnol 102:3765–3777. doi:10.1007/s00253-018-8846-3. 29502180

[17] Krulwich TA, Sachs G, Padan E. 2011. Molecular aspects of bacterial pH sensing and homeostasis. Nat Rev Microbiol 9:330–343. doi:10.1038/nrmicro2549. 21464825 PMC3247762

[18] Wood JM. 2015. Bacterial responses to osmotic challenges. J Gen Physiol 145:381–388. doi:10.1085/jgp.201411296. 25870209 PMC4411257

